# Canine fibroblast growth factor receptor 3 sequence is conserved across dogs of divergent skeletal size

**DOI:** 10.1186/1471-2156-9-67

**Published:** 2008-10-21

**Authors:** Logan B Smith, Danika L Bannasch, Amy E Young, Deborah I Grossman, Janelle M Belanger, Anita M Oberbauer

**Affiliations:** 1 Department of Animal Science, University of California, Davis, CA, USA; 2 Department of Population Health and Reproduction, School of Veterinary Medicine University of California, Davis, CA, USA

## Abstract

**Background:**

Fibroblast growth factor receptor 3 (FGFR3) is expressed in the growth plate of endochondral bones and serves as a negative regulator of linear bone elongation. Activating mutations severely limit bone growth, resulting in dwarfism, while inactivating mutations significantly enhance bone elongation and overall skeletal size. Domesticated dogs exhibit the greatest skeletal size diversity of any species and, given the regulatory role of FGFR3 on growth plate proliferation, we asked whether sequence differences in FGFR3 could account for some of the size differences.

**Methods:**

All exons, the promoter region, and 60 bp of the 3' flanking region of the canine FGFR3 gene were sequenced for nine different dog breeds representing a spectrum of skeletal size. The resultant sequences were compared to the reference Boxer genome sequence.

**Results:**

There was no variation in sequence for any FGFR3 exons, promoter region, or 3' flanking sequence across all breeds evaluated.

**Conclusion:**

The results suggest that, regardless of domestication selection pressure to develop breeds having extreme differences in skeletal size, the FGFR3 gene is conserved. This implies a critical role for this gene in normal skeletal integrity and indicates that other genes account for size variability in dogs.

## Background

Fibroblast growth factor receptor 3 (FGFR3) is a membrane-bound tyrosine kinase receptor that regulates cellular proliferation within the growth plate of long bones. In bone elongation, FGFR3 serves to limit the proliferative activity of the chondrocytes while promoting differentiation and contributing to the mineralization at the chondro-osseus junction [1]. Mice and humans with a gain of function mutation have impaired bone elongation, resulting in achondroplasia [2-4], whereas loss of function mutations result in skeletal overgrowth and severe appendicular abnormalities [5-7].

In mice and humans, the FGFR3 gene contains 19 exons [8] that are differentially spliced to form two distinct isoforms that differ in tissue expression, ligand binding affinity, and cellular response [9]. The two FGFR3 isoforms, IIIb and IIIc, are generated by alternative RNA splicing of exon 7 to either exon 8 or 9. A third isoform, IIIa has been described for other members of the FGFR family but not for FGFR3 [10]. The alternative splicing creates receptors with distinct extracellular binding domains with different ligand binding specificities and differential expression: FGFR3 IIIb is expressed in epithelial cells and FGFR3 IIIc is expressed in mesenchymal-derived cells [6,11]. Mice lacking FGFR3 IIIc display significant skeletal overgrowth, exaggerated limb growth, distorted growth plates indicative of elevated proliferation, and diminished mineralization [6]. Mice lacking the FGFR3 IIIb isoform do not exhibit those skeletal phenotypes [6] indicating the IIIc form as critical for normal skeletal development.

Mutations in the ligand binding domain, the transmembrane domain, or the tyrosine kinase domains have all been associated with constitutive activation and short stature [11]. In contrast, genetically engineered mice that fail to express functional FGFR3 exhibit extreme skeletal overgrowth [12,13]. In sheep, a naturally-occurring mutation in FGFR3 causes inactivation of a kinase domain and results in similar excessive growth [14]. Furthermore, sheep heterozygous for the naturally-occurring loss of FGFR3 function mutation exhibit enhanced frame size without the detrimental skeletal effects associated with two mutant alleles [15]. The reported FGFR3 mutations in humans, mice and sheep arise predominantly from point mutations in the coding regions [16,17]. Depending on the location of a single nucleotide polymorphism (SNP) in FGFR3, "graded activation" of the gene occurs, generating a spectrum of skeletal size [17]. For example, a SNP in the transmembrane domain of FGFR3 commonly results in achondrodysplasia, a specific skeletal disorder that results in short stature and disproportionately short limbs [18]. However, point mutations in the second tyrosine kinase domain of FGFR3 can result in lethal thanatophoric dysplasia or severe achondroplasia with developmental delay and acanthosis nigricans [17,18].

As a consequence of domestication and selective breeding, dogs exhibit the greatest range of skeletal size diversity in any single species. Given the pivotal role of FGFR3 in modulating skeletal size, we asked whether SNPs within the FGFR3 gene could account for height variations seen in the three different Poodle varieties specifically selected on wither height: Toy (less than or equal to 25.4 cm), Miniature (between 25.4 and 38.1 cm), and Standard (greater than 38.1 cm). In addition, the FGFR3 gene was sequenced in several dog breeds defined as chondrodysplastic by Stockard [19]. These breeds exhibit dwarfism as a fixed trait with the entire population of a given breed sharing the same mutation and exhibiting the same altered limb morphology [20,21]. Similarities between human and canine phenotypes suggest a potential role for FGFR3 in different dog breeds displaying dwarfism.

## Methods

The Ensembl FGFR3 gene model was used to define putative exons for the canine FGFR3 gene [22] including the FGFR3 IIIb and FGFR3 IIIc isoforms. The primer pairs for the promoter, the 19 exons comprising FGFR3 IIIb and FGFR3 IIIc, and 60 bases at the 3' end of the FGFR3 gene (Table 1) were developed based on the homology of the canine Ensembl reference Boxer sequence and the published human genome sequence. In many cases the amplicon for a given exon included adjacent intronic sequences. A predicted exon defined in the Ensembl FGFR3 IIIc isoform model, located between exon 4 and 5, was excluded from sequencing because of complete lack of homology to other FGFR3 cDNA transcript sequences (human, mouse, and cow).

**Table 1:** The sequences of the forward and reverse primers flanking the promoter, 19 exons, and 3'flanking sequence for canine FGFR3 (Genbank Accession: EU853457), annealing temperatures, and amplicon sizes

Ensembl Exon	Forward	Reverse	Anneal (°C)	Amplicon (bases)
Promoter^a^	gacgcgtggcctagattc	gagcatgtgcccctgatac	60	699
1 and 2	caaacctcccagaacaggac	cccgcagggatacagtctt	61.5	833
3	cgtgtgcaggtgctcagtat	gtgtcctcagcctcatcctc	60	495
4	cgtgcgtgtgacaggtaaat	ctgcagtacaggtccccaac	59	394
5	accatgtggcttagccttga	tgtttctccacaacgcatgt	59	472
6	ccatctcgtggctgaagaac	gctgtacaccttgcagtgga	58	437
7	acatgcgttgtggagaacaa	taccacttctcccctgatgg	55	399
8 IIIb^b^	cagcatttctgactgcagga	ggctcggaacctggtatcta	58	401
8 IIIc^c^	atgtggactctggctgtggt	cacgagttctgtggagcaag	60	301
9	acacccccttctccattctc	gagtgcagtgcgagtctcag	59	407
10	tggagcctgggttatttgtc	cagtcatcacactgcccatc	59	500
11	aaggttgtggggcaagtatg	ccaggtctgagaggtccttg	59	358
12	aggccatcggtattgacaag	gtacaggggtcttggagcag	60	349
13	tccttcctgcacagatgatg	aagctcccaagtggtcctg	58	461
14	ttctccctccccccccttccccagac	tcccgagggcaggggcccttgtc	59	350
15	cagccaggccctggctgccgccac	agggcacctggccgtcaacatgc	59	394
16	accgagtctacacccaccag	acaatgcctcccatgacc	59	426
17	atggacaagccagccaact	ccgacaggtccagatactcc	60	401
18 and 3' flanking	gcagctagtggaggatctgg	cacaccaccagcagcatagt	60	361

a. Amplicon represents the putative promoter region

b. Exon indicating the FGFR3 IIIb isoform

c. Exon indicating the FGFR3 IIIc isoform

All amplicons were sequenced for three to eight dogs of each of the three Poodle varieties, and, representing chondrodysplastic breeds, a single Basset hound, Dachshund, Pekingese, Cardigan Welsh Corgi, and Pembroke Welsh Corgi were sequenced. A Nova Scotia Duck Tolling Retriever and Chesapeake Bay Retriever, both affected with short stature, curved limb skeletal dysplasia, were also sequenced as was a Dalmatian that exhibited no skeletal abnormalities. All dogs sequenced were registered purebreds with the American Kennel Club with the Poodles unrelated at the grandparent level. The Nova Scotia Duck Tolling Retriever and Chesapeake Bay Retriever were diagnosed as affected with chondrodysplasia by x-ray. Buccal swab-derived DNA was extracted for each Poodle as previously described [23] and then subjected to PCR amplification using primers specific for the regions of interest. The 20 μL PCR reactions contained 0.5 U Amplitaq Gold DNA Polymerase (Applied Biosystems, Foster City, CA, USA), 200 μm dNTPs (Applied Biosystems), 1.5 mm MgCl_2 _(Applied Biosystems), 1 × Geneamp PCR Gold buffer (Applied Biosystems); 0.2 μm of each forward and reverse oligonucleotide primer (Operon Biotechnologies, Huntsville, AL, USA), and 10–20 ng template genomic DNA. Reaction mixtures were subjected to a thermal cycling program of 94°C for 12 min, followed by 35 cycles of 94°C for 30 s, 45 s at the annealing temperature (see Table 1), and 72°C for 45 s and a final elongation stage of 72°C for 10 min were completed. Blood samples for the other breeds were collected from patients of the University of California, Davis, Veterinary Medical Teaching Hospital. DNA was extracted using the QiaAmp Blood Mini Kit (Qiagen) and PCR amplified as described. The amplified products for each region for each dog were gel purified with a gel-purification kit (Qiagen, Valencia, Calif.) and sequenced at an automated DNA sequencing facility (Davis Sequencing Inc, Davis, Calif.). Alternatively, some amplified products were purified with the QiaQuick PCR Purification Kit (Qiagen), sequencing reactions carried out using the Big Dye Terminator v3.1 cycle sequencing kit (Applied Biosystems), and products processed on an ABI 3100 Genetic Analyzer.

The derived sequences for each exon and the flanking promoter and 3'end were aligned with the reference Boxer sequence (Vector NTI Advance, version 10.3.0, Invitrogen Corp, Carlsbad Calif). Any potential SNPs in the sequences were confirmed or rejected by sequencing the complementary strand as well as sequencing additional dogs of the breed that exhibited the SNP. The 699 bp upstream of exon 1 was analyzed for the presence of promoter elements using the Genomatix [24] software tools Gene2Promoter and MatInspector [25].

## Results

Remarkably, Poodles of all three varieties that differed in skeletal size had fully conserved FGFR3 sequence across all 19 exons, the promoter region, and the downstream 3' flanking sequence. Looking across breeds that exhibit extreme skeletal morphologic differences also revealed 100% exon sequence conservation relative to the published reference Boxer sequence. As predicted by Ensembl, there are 17 introns for the canine FGFR3 gene. The primers used for sequencing the exons also included complete intron sequencing for ten of those introns and partial coverage for the remaining seven. Based upon the predicted sizes of the introns, partial sequence coverage ranged from a high of ~99% of the intronic region sequenced to a low of just over 15% for the largest introns. The only sequence variation observed relative to the reference Boxer sequence, across all dogs evaluated, was in intronic regions. Single SNPs were identified within introns 1, 8, 10, 12, and 16 for a total of 5 SNPs (Table 2). The identified intronic SNPs were sporadic and not present in all the dogs exhibiting a common phenotype.

**Table 2:** Location and number of sequenced single nucleotide polymorphisms (SNPs) identified within canine FGFR3 introns^a^

Intron	Location^b^	SNP^c^	SNP frequency^d^
1	541	C > T	1 of 18
8	400	C > T	31 of 34
10	22	G > T	4 of 33
12	11	C > T	21 of 37
16	35	G > A	1 of 20

a. The intronic SNPs were not present in all the dogs exhibiting a common phenotype

b. Number of bases from intron start

c. Typical change relative to reference Boxer genome

d. Number of dogs with SNP relative to total number of dogs sequenced

Analysis of the 699 bp region upstream of exon 1 using the Gene2Promoter software indicated that no classical promoter elements were apparent although MatInspector identified several possible transcription factor elements within the promoter region sequenced. The most notable transcription factor elements included two CCAAT box promoter elements, a GA box, three cAMP response elements, three GC box Sp1-binding sites, two RXR heterodimer binding sites, and two estrogen response elements. Additionally, two Prox1 binding sites were identified in the intron between exons 1 and 2.

## Discussion

In addition to differential skeletal size observed in sheep carrying mutations in FGFR3, greater than 95% of human dwarfism achondrodysplasia cases are caused by a gain-of-function mutation in FGFR3 [4]. Thus, the FGFR3 gene is the foremost candidate gene for regulating the diversity of skeletal size observed in dog breeds. The lack of sequence divergence in the FGFR3 gene or its regulatory regions across all the dogs and dog breeds assessed in this study suggests that selection pressure to maintain this precise sequence remains very strong. The conservation of this gene persists even in the face of human breeding schemes and breed development as seen by the absence of sequence mutations within the coding region of the gene across all the diverse dog breeds analyzed. Rare and only minor sequence divergence was observed for intronic regions. The intronic SNPs identified were deemed neutral as they were not associated with a common phenotype or height category. Due to the homozygosity present in breeds exhibiting fixed traits, it was unnecessary to sequence additional individuals from each breed.

Achondroplasia in humans is most frequently due to constitutive activation mutations within the transmembrane domain of FGFR3 [2]. However, when Martinez et al., [26] sequenced only the FGFR3 transmembrane domain, they found no difference in that sequence when comparing achondroplastic dogs to a non-achondroplastic dog, the German shepherd. Likewise, dwarfism in Dexter cattle is not due to mutations in the transmembrane domain of FGFR3 [27]. These findings, coupled with the current results, imply the essentiality of the FGFR3 gene in normal skeletal development. Spontaneous inactivating or genetically engineered null mutations of the FGFR3 gene result in appendicular abnormalities, enhanced proliferation of growth plate chondrocytes, and reduced cortical bone thickness [6,28]. Furthermore, lifespan is reduced in genetically FGFR3 null mice [29].

The FGFR3 protein and mRNA show a high degree of sequence homology across divergent mammalian species. For example, a pairwise alignment of the dog, human, and mouse FGFR3 protein sequences exhibits a 96% homology for consensus of amino acid pairs among all ungapped positions in the IIIb isoform. Similarly in the dog FGFR3 IIIc isoform, a pairwise alignment to the human and mouse protein sequence results in 95% and 94% homology, respectively. As expected, when the sequenced dog FGFR3 genomic exons were compared with published dog ESTs (mRNA) they were identical and when aligned with human and mouse ESTs exhibited homologies similar to human and mouse protein alignments as described above [30].

Expression of FGFR3 has been proposed to be the essential link to coordinate growth plate cell proliferation with the osteogenesis and mineralization necessary to structurally support the elongating skeleton [6]. Disruption of this coordination could jeopardize the integrity of the skeleton in terrestrial species. The pathology in animals with gain of function mutations likewise exhibit dysregulation between cellular proliferation at the growth plate and ossification [5]. Thus, the selection pressure to maintain a coordinated balance between bone elongation and ossification apparently outweighed any human selection pressure to develop dog breeds with distinct morphologies.

Recent reports have identified a QTL association between IGF-I and skeletal size in the dog [31], reiterating the association between IGF-I and growth in livestock [32-34]. The IGF-I gene has a more expansive set of target tissues when compared to the restrictive targets of FGFR3. The systemic effects of IGF-I, with its multitude of actions, would be subject to less stringent selection pressure than a gene that balances structural support with overall size. Therefore, genetic change in the IGF-I locus could be tolerated whereas sequence change in FGFR3 would not be permitted.

Although a classical promoter was not identified by the sequence analysis of the dog FGFR3 promoter region, many genes utilize diverse core promoters that vary from the standard TATA-dependent promoters (reviewed in [35]) including the murine FGFR3 [10]. The in silico FGFR3 promoter analysis did underscore the similarities and differences between dogs, mice, and humans. A pairwise alignment of the dog FGFR3 promoter region with mouse, and human resulted in a poor identity (52% and 49%, respectively). Nevertheless, the key functional transcriptional enhancer elements characterized in the mouse and human FGFR3 gene, such as cAMP response elements, SP1, and Prox1 binding sites [36,37], were identified in the canine FGFR3 promoter region. This suggests common regulation of the expression and function of the FGFR3 gene. Interestingly, potential estrogen binding domains were identified within the dog FGFR3 promoter region. Estrogen is known to play a major role in growth plate arrest and ossification [38]. Although the mechanism used in growth plate closure is unknown, the presence of estrogen binding domains suggests a mechanism for estrogen regulation of chondrocyte activity through FGFR3 during skeletal maturation [39].

## Conclusion

The present study revealed homogeneity across many different breeds of dogs, indicating the essentiality of the FGFR3 gene product in maintaining skeletal integrity. Deviations from the consensus sequence appear to be quickly subjected to purifying selection in purebred dog breeds such that they are not maintained.

## Authors' contributions

LBS participated in carrying out the study, sequencing coordination, sequence alignment and in drafting the manuscript. DLB participated in conceiving the study and in the study design. AEY participated in carrying out the study, sequencing, and sequence alignment. DIG and JMB participated in sequencing and sequence alignment. AMO participated in conceiving the study, study design, coordination, and in drafting the manuscript.
